# Considering Patients' Mental Capacity When Giving Them Bad News May Help Their Well-Being: A Case of Suicide Attempt after Being Informed of Lung Cancer Diagnosis

**DOI:** 10.1155/2014/645769

**Published:** 2014-05-21

**Authors:** Toshiyuki Kobayashi, Satoshi Kato, Mitsuo Takeuchi

**Affiliations:** ^1^Department of Psychiatry, Jichi Medical University, 3311-1 Yakushiji, Shimotsuke-shi, Tochigi 329-0498, Japan; ^2^Jiundo Hospital, 4 Chome-14-53 Sekimachiminami, Nerima, Tokyo 177-0053, Japan

## Abstract

Mental capacity is a central determinant of patients' ability to make autonomous decisions about their care and deal with bad news. Physicians should be cognizant of this when giving patients bad news in efforts to help them to cope with the illness and to avoid a deterioration of their mental well-being. To show the importance of this concept, a case of suicide attempt with lung cancer is exemplified. A 76-year-old woman attempted suicide after receiving a diagnosis of lung cancer. Her recent life had been emotionally turbulent and she did not have sufficient mental capacity to accept and cope with this truth. She developed depression before attempting suicide.

## 1. Introduction

Suicide is a recognized social problem in Japan, and the number of suicides has exceeded 30,000 for many years. The number one cause of death in Japan is malignant neoplasm [1] and receiving such a diagnosis can often precipitate a life crisis and cause psychiatric problems including depression and anxiety disorder. In the last few decades in Japan, many clinicians have come to believe that patients with malignant tumors must be told of their illness, in line with the principles of receiving informed consent from patients to undergo certain diagnostic procedures and decide among treatment options. This has meant that giving bad news has become a regular task for physicians; in fact, it could be said that this is often done routinely.

In this report, the authors present the case of a patient who attempted suicide after her physician informed her of incurable malignancy and transferred her care. The authors discuss the need for physicians to be cognizant of patients' mental capacity when giving them bad news in efforts to help them to cope with the illness and avoid a deterioration of their mental well-being.

## 2. Case Report

A 76-year-old woman who threw herself in front of a train arrived at our hospital by ambulance. She had landed between the rails and evaluation in the emergency room found no injuries except a small subcutaneous hematoma in the left temporal region. When chest X-ray examination and brain computed tomography (CT) revealed she had lung cancer with brain metastasis, the emergency room staff referred her to psycho-oncology. Following a diagnosis of depression, she was moved to the psychiatric ward.

The patient was the daughter of a farmer. In the chaotic times after World War II, she unwillingly got married, divorced a year later, and worked in various jobs before eventually opening a small bar. She did not have family history or past history of suicide or depression. After running the bar for more than 20 years, in 2002 at the age of 70, she handed running the bar over to her employee. The next year, Mr. A, a customer of the bar and her friend for 30 years who was a widower and 4 years older than her, began to make advance towards her, stating his wish to live with her and to guarantee her an adequate income. Issues of money and marriage were of no importance to her, but she began to dream of life with a reliable partner in her golden years.

When she was admitted to hospital with severe hypertension in 2004, Mr. A was unexpectedly cold towards her and she became suspicious of his sincerity towards her. After discharge, repeated episodes of hypertension led to emergency admissions. In 2005, tired of a single life, anxious about the future, and led on by Mr. A's ardent desire to be with her, she decided to live with him. However, until she moved into Mr. A's house she did not know that his socially withdrawn son, who was in his fifties, would be living with them. She frequently witnessed family quarrels and Mr. A used to go out early in the morning to avoid quarreling, leaving her with his son. The son followed what she did closely all day, became extremely angry when he did not get his way, and was sometimes violent towards her. She endured the situation for about a year until she developed insomnia, avolition, and deteriorated hypertension. She moved in with her nephew for 6 months to escape the situation and her health improved. As she felt recovered and Mr. A demanded she return because her absence was an “inconvenience,” she decided to move back in with them again.

**Figure 1:**
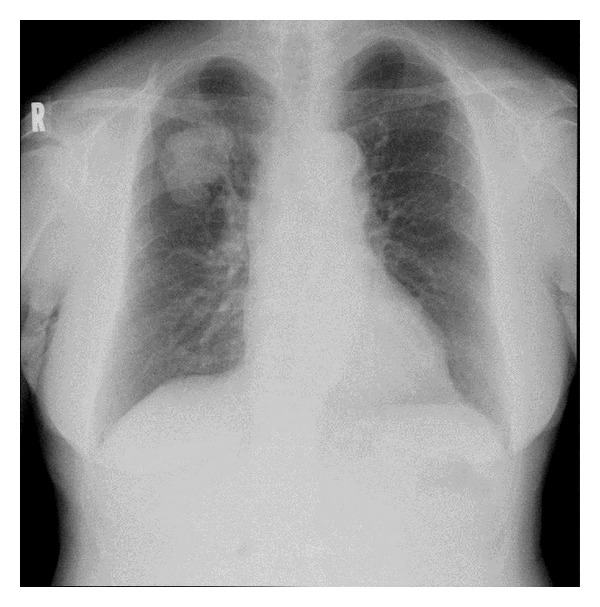
Routine chest X-ray examination shows a large coin lesion in the right upper lung field.

However, the son treated her ever more unkindly, claiming she had designs on his father's assets. With this and having an unreliable partner, she started to feel uncertain about the future and again developed insomnia and avolition as well as loss of appetite in 2008. In March that year, a routine chest X-ray examination revealed a large coin lesion in the right upper lung field (Figure 1) and she was referred to a general hospital. When she told Mr. A of the shadow on her lung, he told her to move out. She managed to get him to let her stay until a detailed examination was carried out. At this time she began to have suicidal ideation.

After the detailed examination was performed in April, she was diagnosed with squamous cell carcinoma of the lung with metastasis to the supraclavicular lymph nodes, right adrenal gland, and liver. She wanted to be informed of the actual nature of her illness and said she did not need relatives present when the doctor explained it to her. She was informed she had advanced lung cancer that was considered nonoperable and not treatable by chemotherapy or radiation therapy. The physician recommended she visit a local hospital, because he had no therapeutic options. The next day, she moved to her nephew's house with 50,000 yen “palimony” from Mr. A. She suffered insomnia and had suicidal ideation.

Three days after receiving the bad news, she got on a local train headed south to find a distant place to die. However, she could not find a suitable place before reaching the end of the line, so she traveled north by Shinkansen. She got off at a spa town with a suspension bridge nearby and took a taxi to the suspension bridge. However, she abandoned the idea as it was already dusk and returned to the Shinkansen station by bus. From there, she took a local train to the closest station to her nephew's house. She suddenly thought to throw herself in front of a train there. She was brought to our hospital after this suicide attempt.

**Figure 2:**
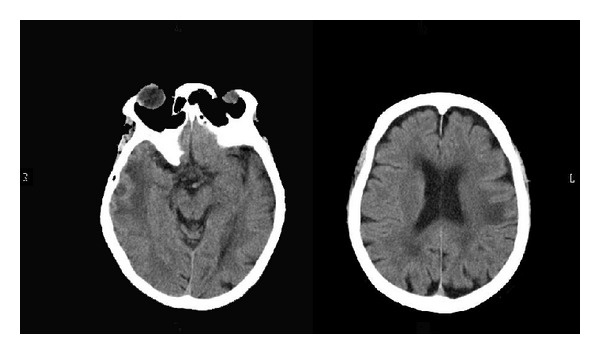
Brain CT performed during emergency care revealing a low density area with a nodular structure in the lower part of the right temporal lobe and a smaller low density area in the left paraventricular subcortical area suggestive of brain metastasis and brain edema.

The psycho-oncologist started her on fluvoxamine (50 mg) and zolpidem (5 mg). Upon admission to the psychiatric ward, she did not seem so depressed although she talked much about her disillusionment and disappointment with Mr. A. Brain CT performed during emergency care had revealed a low density area with a nodular structure in the lower part of the right temporal lobe and a smaller low density area in the left paraventricular subcortical area which suggested brain metastasis and brain edema (Figure 2). However, her responses were natural and prompt, she talked logically, and her behavior was polite and appropriate. There seemed to be no manifestations of the brain metastasis. Psychometric tests such as the Wechsler Adult Intelligence Scale were not performed as it seemed ungracious to perform further evaluation of a dying patient who had survived suicide. While on the ward she was informed of her illness once again with her sister present and she expressed the wish to spend the rest of her life in hospital. We planned on transferring her to a hospital near her sister's house when her mental stability was confirmed.

Around 4 weeks later she came to talk much, especially in the morning, and appeared to be hypomanic. She was started on sodium valproate 200 mg, which resolved the hypomania. However, she was still euphoric and said repeatedly how happy she was to live in such a nice place. Around 6 weeks later her responses were incoherent and circumstantial. She laughed inappropriately and sometimes fell to the floor. Brain CT revealed enlargement of brain edema. She was transferred to a hospital near her sister's house on hospital day 69. She died about 8 weeks after transfer.

## 3. Discussion

Telling bad news or truth-telling is crucial but problematic practice in medicine, especially in oncology. It is highly relevant for clinical practice ranging from decision making for treatment option to palliative care [2]. There remains a strong resistance against disclosure of diagnosis and prognosis in terminally ill patients, because of the fear of causing despair to patients [3]. Although a protocol for breaking bad news was proposed [4, 5], disclosure practices are far from being the norm in many countries [3], and most of the physicians lacked the essential knowledge and skills for breaking bad news [6]. Having a cancer causes psychological problems and cancer patients have higher suicide risk [7–9]. It is of no wonder how unskilled truth-telling may make the patients' psychological problems more serious.

On the other hand, if the patients have great resilience, they would overcome even the impact from unskilled truth-telling. But most of the physicians lacked the experience of estimating the patients' mental capacity. Unless patients show obvious signs of mental or cognitive disorder, clinicians typically do not explicitly assess their mental capacity to be suitably informed and make autonomous decisions [10].

The present patient faced these two difficulties: her physician's unskilled truth-telling and thoughtlessness of her mental capacity.

The patient's physician told her that she had an incurable illness and recommended that she visit a local hospital. At that time, she was depressed by Mr. A's unreliability and her feelings of being abandoned by him. She may well have felt the same sense of abandonment when her physician recommended she go to another hospital. Her depressed mood might have deteriorated from here to the point of impulsive suicide attempt.

This case clearly illustrates that the patient's mental capacity should have been evaluated before telling her the bad news. Mental capacity is a multidimensional construct that is a central determinant of an individual's ability to make autonomous decisions [11]. Grisso et al. proposed a “four abilities” model of patients' mental capacity to make treatment decisions, comprised of understanding, appreciation, reasoning, and ability to express a choice about treatment [12]. Mental capacity is variously defined and there are no easy tools to assess it. The Mental Capacity Act of England and Wales suggests that a patient does not have capacity if there is an impairment of or disturbance in the functioning of brain or mind that causes difficulty in decision making because the individual (1) is unable to understand information relevant to the decision, (2) cannot retain the relevant information, (3) is unable to use this information as part of the decision-making process, or (4) cannot communicate the decision [10]. However, in the clinical setting where doctors must evaluate their patients' mental capacity through clinical interview, making judgments according to such criteria is likely to be difficult.

Mental capacity has always been an important issue in psychiatry, especially regarding the use of compulsion in the care of patients with severe communication difficulties or cognitive problems and patients with psychotic disorders [11]. Needless to say, telling such patients they have cancer will create problems because their mental capacity is impaired. Yet, this recognition should be extended to all cancer patients, in line with the principles of informed consent which require clinicians to tell patients their detailed clinical findings. Physicians must often give detailed information to patients and give it to them quickly because cancer treatment generally should be started as early as possible, and they need the patient's treatment decision as quickly as possible. Such prompt action not only benefits the patient but also avoids medical malpractice litigation; however, the patient's mental capacity should still be carefully considered at this time.

To date, impairment of mental capacity in heterogeneous groups, such as acutely medically ill patients in hospital, has not been extensively studied [10]. In a study of the prevalence of mental incapacity among inpatients admitted to two acute general medical wards at a London teaching hospital, Raymont et al. found that 24% (72/302) of patients were automatically assigned to the incapacity group because of being severely cognitively impaired, unconscious, or unable to express a choice, 24% (*n* = 71) refused to participate or could not speak English, and of the remaining 159 patients, 31% were judged not to have mental capacity [10]. They found that the factors associated with mental incapacity were increasing age and cognitive impairment, a finding suggesting that large numbers of patients with mental incapacity will be encountered in general medical practice.

From another angle, telling patients bad news like they have cancer is a significant speech act [13, 14] and can be traumatic for some more than others. A longitudinal investigation by Gonçalves et al. [15] of posttraumatic stress disorder (PTSD) in 121 patients with ovarian cancer revealed that 36% of the total sample had symptoms of PTSD at the time of starting chemotherapy treatment, with the traumatic experience inferred to be the bad news they received about their cancer. Being told one has incurable cancer is not a process of merely receiving and processing the information imparted—it can be just like receiving a death sentence or, when the cancer is asymptomatic, suffering a surprise attack. For our patient, being informed of her illness was both a death sentence and a surprise attack: her lung cancer was asymptomatic and incidentally discovered on a routine chest X-ray examination during treatment for hypertension, and it was already advanced with no treatment options available to her. Receivers of such information like this require a larger mental capacity to bear the impact of what they are being told. Therefore, in some cases, not telling patients with mental incapacity about their malignancy can be justified.

The patient's physician did not understand her situation and did not evaluate her mental capacity before informing her of the bad news. He presumed that she had sufficient mental capacity from her nature. This presumption seems reasonable at first glance, given that she seemed to be an independent person who had been running her business alone and despite already having brain metastasis she showed no apparent mental impairment. However, the considerable problems she had in her life with Mr. A might have reduced her mental capacity.

It may well be difficult to get a detailed life history for patients in the internal medicine setting and evaluate their mental capacity. Most of the physicians are unused to ask about patients' mental states, not to mention risk factors of suicide.

The lesson learned from the case is that interview about braking bad news requires careful setting up and step-by-step procedure addressing reducing the patient's distress [4], even if the patient seems to have enough mental capacity. If the physician does not have a plenty of time and essential skills for breaking bad news, there is a simple way to enlarge the patient's mental capacity: involving significant others [4]. Physicians should consider the need for family members or other important persons to be present when informing them. Although involving others should be the patient's choice [4], the physician should strongly recommend it. It may support patients with sharing news of their cancer diagnosis with their important persons [16]. The present patient initially wanted to be informed of her illness alone. After the admission, involvement of her sister seemed to restore her mental balance. It is crucial to let patients know that there are definitely companions who will walk with them.

It was indeed unfortunate that our patient attempted suicide and that her time after surviving the attempt was short. However, hospitalization seemed to contribute her well-being and her time on the ward was stable and happy, even if the happiness possibly originated in part from the euphoria due to brain metastasis. She could find companions, including her sister and the ward staffs, who were walking with her.
